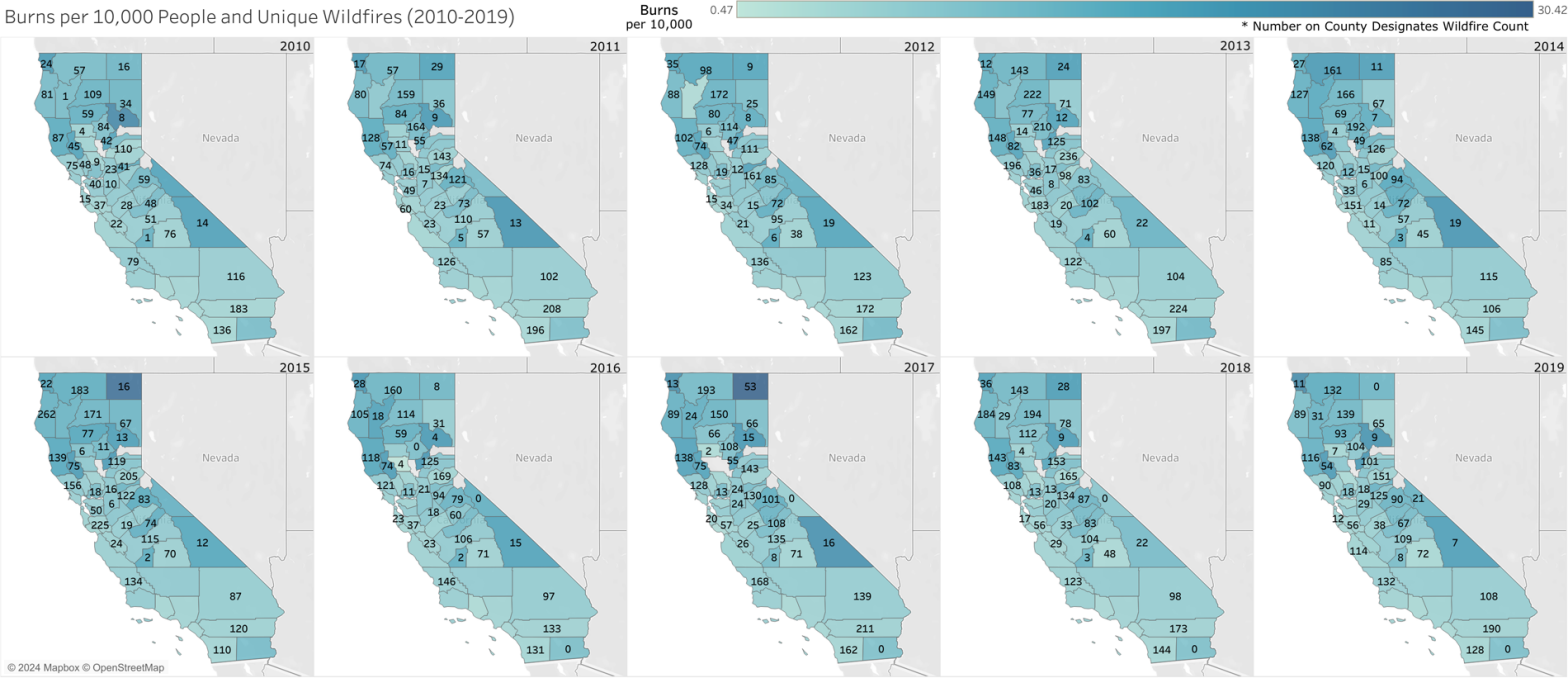# 961 The Impact of State Wildland Fires on Burn Related Injuries

**DOI:** 10.1093/jbcr/iraf019.492

**Published:** 2025-04-01

**Authors:** Devi Lakhlani, Clifford Sheckter

**Affiliations:** Santa Clara Valley Medical Center; Santa Clara Valley Medical Center

## Abstract

**Introduction:**

Wildland fires present a threat to society on many fronts including human morbidity. The relationship between wildland fires and burn related morbidity remains poorly characterized. Further, fluctuations in fire seasons and fire source/mechanism creates uncertainty with respect to effects on human health. We aim to characterize the effects of wildland fires on burns treated in one state.

**Methods:**

All burns treated in emergency departments (ED) were extracted from the State’s Department of Health Care Access and Information database, 2010-2019. Injury totals were summarized at the county level per year. This data was merged with county level data from the State Department of Forestry and Fire Protection. Rural, urban, or suburban designations were from the State Association of Counties. The number of unique fires, total acres burned, and source of fire were collated at the county level per year. The proportion of wildland fires caused by each mechanism was used as a predictor to model the relative contribution of each mechanism to burn injuries, accounting for the variability in total wildfire activity across rural counties. Linear regression and mixed-effects modeling were performed for rural counties.

**Results:**

458 county-year observations were included, with rural counties comprising 224 (48.9%). The median number of burns per county/year was 53.0 (IQR 27.75, 108.0). Rural counties showed significant fluctuations in burn incidence by year compared to stable burn incidence in urban counties. In contrast, the number of wildfires per county varied greatly in both urban and rural counties. The number of unique wildfires averaged 3,092 ± 399.0 across 10 years, with 2010 and 2013 exhibiting the smallest (2,331) and largest (3,662) number of total wildfires. Total burns per county-year was positively associated with total wildfires (coef 0.34, p <.001), but not total acres burned (p=0.466). Mechanisms most positively associated with burns included smoking (coef 10.48, p=0.002), playing with fire (coef 8.80, p=0.002), campfires (coef 6.51, p <.001), vehicles (coef 4.27, p <.001), and arson (coef 3.52, p <.001). A sensitivity analysis using a mixed-effects model with random intercepts for rural counties, accounting for inter-county variation, revealed the mixed-effects model improved predictive efficiency for total burns (AIC: 1861.55 < 2347).

**Conclusions:**

Rural counties experienced great fluctuation in wildland fire frequency and size, though only the frequency of fires was associated with ED visits for burns. Multiple mechanisms of fire were associated with burns, most notably smoking-related fires had the largest effect.

**Applicability of Research to Practice:**

Burn systems in this state should be prepared to care for injuries resulting from wide fluctuations in frequency of wildland fires. Prevention efforts are needed for many of the mechanisms of wildland fire including smoking.

**Funding for the Study:**

N/A